# Pediatric emergency department visits and ambient Air pollution in the U.S. State of Georgia: a case-crossover study

**DOI:** 10.1186/s12940-016-0196-y

**Published:** 2016-11-25

**Authors:** Qingyang Xiao, Yang Liu, James A. Mulholland, Armistead G. Russell, Lyndsey A. Darrow, Paige E. Tolbert, Matthew J. Strickland

**Affiliations:** 1 Department of Environmental Health, Rollins School of Public Health, Emory University, Atlanta, GA USA; 2 Department of Civil and Environmental Engineering, Georgia Institute of Technology, Atlanta, GA USA; 3 School of Community Health Sciences, University of Nevada – Reno, 1664 N Virginia Street MS 0274, Reno, NV 89557 USA

**Keywords:** Air pollution, Pediatric Emergency Department Visits, Multipollutant model, CMAQ

## Abstract

**Background:**

Estimating the health effects of ambient air pollutant mixtures is necessary to understand the risk of real-life air pollution exposures.

**Methods:**

Pediatric Emergency Department (ED) visit records for asthma or wheeze (*n* = 148,256), bronchitis (*n* = 84,597), pneumonia (*n* = 90,063), otitis media (*n* = 422,268) and upper respiratory tract infection (URI) (*n* = 744,942) were obtained from Georgia hospitals during 2002–2008. Spatially-contiguous daily concentrations of 11 ambient air pollutants were estimated from CMAQ model simulations that were fused with ground-based measurements. Using a case-crossover study design, odds ratios for 3-day moving average air pollutant concentrations were estimated using conditional logistic regression, matching on ZIP code, day-of-week, month, and year.

**Results:**

In multipollutant models, the association of highest magnitude observed for the asthma/wheeze outcome was with “oxidant gases” (O_3_, NO_2_, and SO_2_); the joint effect estimate for an IQR increase of this mixture was OR: 1.068 (95% CI: 1.040, 1.097). The group of “secondary pollutants” (O_3_ and the PM_2.5_ components SO_4_^2−^, NO^3−^, and NH^4+^) was strongly associated with bronchitis (OR: 1.090, 95% CI: 1.050, 1.132), pneumonia (OR: 1.085, 95% CI: 1.047, 1.125), and otitis media (OR: 1.059, 95% CI: 1.042, 1.077). ED visits for URI were strongly associated with “oxidant gases,” “secondary pollutants,” and the “criteria pollutants” (O_3_, NO_2_, CO, SO_2_, and PM_2.5_).

**Conclusions:**

Short-term exposures to air pollution mixtures were associated with ED visits for several different pediatric respiratory diseases.

**Electronic supplementary material:**

The online version of this article (doi:10.1186/s12940-016-0196-y) contains supplementary material, which is available to authorized users.

## Background

Associations between ambient air pollution concentrations and human health responses have been reported in numerous epidemiological and experimental studies [1–3]. Children are a vulnerable subpopulation due to their developing physiology and frequent outdoor activities [4, 5]. Associations between single air pollutant concentrations and pediatric health outcomes have been reported in several previous studies; however, children are exposed to a mixture of air pollutants, and given that the composition and correlation of air pollutants varies in time and space, there is no single pollutant that can act as a universal indicator of a specific air pollution mixture [6].

In recent years, the field has seen growth in the number of methodological approaches used to estimate the joint health effects of multiple air pollutants [7, 8]. A recent review of studies that implemented multipollutant exposure metrics to estimate health effects of ambient air pollution reported that although multipollutant exposure metrics were limited by the lack of ‘gold standard’, these approaches have been useful for characterizing multipollutant exposures [9]. Various methodologies have been advanced to develop multipollutant exposure indexes/metrics to assess multipollutant health effects. For example, Stieb et al. [10] and Szyszkowicz [11] developed air quality health indexes based on combinations of air pollutants’ short-term health associations. Coull et al. [12] applied Bayesian kernel machine regression that included a hierarchical variable selection function to estimate complex multipollutant health effects and identify important mixture components. Pearce et al. [13] used self-organizing maps to categorize multipollutant day types based on ten air pollutant concentrations and estimated the associations between multipollutant exposure and pediatric asthma ED visits. These studies avoided the testing of complex interactions between air pollutants in epidemiological models, which are limited by statistical power. However, the clustering processes do not make use of information regarding the source and distribution of air pollutants. Winquist et al. [14], Suh et al. [15], and others have grouped air pollutants by their sources and properties to estimate the joint health effects of pollutant combinations using multipollutant models.

High-resolution spatiotemporal estimates of air pollutant concentrations are needed to characterize air pollutant mixtures over time and space. Ground-based air quality monitoring networks provide the most accurate measurements but have limited spatial coverage, and some stations do not measure pollutants continuously. Different air pollutants are measured at different locations, with varying frequency, and with various instruments. These differences can result in measurement errors that vary across pollutants and these errors can bias health risk estimates [16]. In contrast, chemical transport models provide air pollutant concentration simulations at fine-scale resolution with complete coverage in space and time but may introduce uncertainty and bias due to limitations in source data as well as chemical and physical mechanisms. In response to the demand for high-quality air pollution estimates, statistical approaches have been developed to combine the ground measurements and model simulations. A novel data fusion method presented by Friberg et al. [17] blended the ground measurements of twelve air pollutants with simulations from the Community Multi-Scale Air Quality (CMAQ) model in the U.S. state of Georgia and provided daily air pollutant concentration estimates at 12 km resolution from 2002 to 2008. In this study, we used these high resolution air pollution data to estimate associations between ambient air pollution mixtures and pediatric emergency department (ED) visits in Georgia.

## Methods

### Data

#### Health data

Individual-level ED visits for children aged 0–18 years were obtained from the Georgia Hospital Association from January 1, 2002-December 31, 2008. International Classification of Diseases, 9th revision (ICD-9) codes were used to define health outcomes. Case definitions were based on primary ICD-9 codes for asthma or wheeze (ICD-9 codes 493 and 786.07), bronchitis (490 and 466.0), pneumonia (480–486), otitis media (381 and 382), and URI (460–465 and 477). We excluded children younger than age 2 years from the asthma or wheeze group because of challenges in diagnosing asthma in young children [18]. The date of the ED visit and the ZIP code (n = 742) of the patient’s residence were included in the dataset.

#### Air pollutant concentration estimates

Daily air pollutant concentrations at 12-km spatial resolution in Georgia during 2002–2008 were estimated from CMAQ model simulations and ground-based measurements using the approach developed by Friberg et al. [17]. Air pollutants of interest were 1-h maximum carbon monoxide (CO), nitrogen dioxide (NO_2_), and sulfur dioxide (SO_2_); 8-h maximum ozone (O_3_); and 24-h average particulate matter with an aerodynamic diameter of 10 μm or less (PM_10_), particulate matter with an aerodynamic diameter of 2.5 μm or less (PM_2.5_), and PM_2.5_ components sulfate (SO_4_^2−^), nitrate (NO_3_^−^), ammonium (NH_4_^+^), elemental carbon (EC), and organic carbon (OC). This approach fused CMAQ predictions and ground-based measurements of air pollutant concentrations based on their temporal and spatial trends to provide non-missing air pollutant concentration estimates over the study region. Friberg et al. reported that the accuracy, defined as the percentage of spatio-temporal variance in ground-measured pollutant concentrations that was captured by the fusion pollutant concentration estimates in the comprehensive ten-fold cross-validation, for each pollutant was CO (53%), NO_2_ (69%), SO_2_ (14%), O_3_ (88%), PM_10_ (59%), PM_2.5_ (76%), and PM_2.5_ components SO_4_^2−^ (81%), NO_3_^−^ (57%), NH_4_^+^ (72%), EC (53%), and OC (54%) [17]. SO_2_ concentrations had the most error due to the limited number of SO_2_ monitors and because of the challenges of modeling coal combustion SO_2_ plume ground-level impacts in CMAQ. Small negative pollutant concentration estimates were allowed, even though they lack physical meaning, because these negative values represent low air pollution in the overall distribution. Each ZIP code was assigned air pollution estimates based on the 12-km air quality model centroids that fell in the ZIP code area. An unweighted average of pollutant concentrations was calculated when a ZIP code contained more than one centroid. If no air quality model centroid was in the ZIP code then the centroid closest to the ZIP code was used to assign air pollutant concentrations.

Daily temperature and humidity data at 1/8° (approximately 12.5 km) spatial resolution were obtained from the North American Land Data Assimilation System [19, 20]. The meteorological data were aggregated to the ZIP code level using the same approach described above.

### Epidemiological models

Single-pollutant conditional logistic regression models, matched by ZIP code, day-of-week, month, and year, were fit using SAS (SAS version 9.4; SAS Institute Inc., Cary, NC) to estimate associations between 3-day moving average ambient air pollutant concentrations (lags 0-1-2) and daily ED visits. Using a time-stratified case-crossover model, each ED visit was matched with control days on the same day-of-week and in the same month and year. The case-crossover method controls by design for all subject characteristics that do not vary within the month-long reference time windows [21]. Time-varying covariates included in the model were cubic polynomials for 3-day moving average temperature and 3-day moving average humidity; an indicator for warm season (May-October) vs cool season (November-April), holiday, and lag holiday (indicating whether one of the previous 2 days was a holiday); and product terms between the warm season indicator and the cubic polynomials for temperature, humidity, and day-of-season. Cubic polynomials for day of warm (or cool) season (1,…,184) were also included in the model to control for within-month trends of ED visits due to children activities e.g., back to school [4, 22]. Concentration-response was assumed to be linear on the logit scale, and odds ratios (OR) are presented for one interquartile range (IQR) increase in 3-day moving average ambient air pollution concentrations.

For the multipollutant models, we implemented a grouping and modeling strategy described by Winquist et al. [14]. We selected four combinations of air pollutants based on pollutant properties or sources and a fifth combination comprised of criteria air pollutants (except for lead) set by the US National Ambient Air Quality Standards. These five combinations include “oxidant gases” (O_3_, NO_2_, and SO_2_), “secondary pollutants” (O_3_, SO_4_^2−^, NO_3_^−^, and NH_4_^+^), “traffic pollutants” (CO, NO_2_, EC, and OC), “coal combustion pollutants” (SO_2_ and SO_4_^2−^), and “criteria pollutants” (O_3_, CO, NO_2_, SO_2_, and PM_2.5_). We modified the previous groupings [14] by including organic carbon in the “traffic pollutants” combination because some OC comes from motor vehicle emissions and because OC concentrations were well-correlated with EC concentrations in our data (r = 0.71). All multipollutant models included the same set of covariates as the single-pollutant models as well as the 3-day moving average concentrations of each pollutant within the specified combination. The joint effect estimates for IQR increases in the 3-day moving average concentrations of all pollutants within the combination were estimated from the multipollutant models. We also fit multipollutant models with first-order multiplicative interaction terms between the pollutants, and we estimated the joint effects for IQR increases in the 3-day moving average concentrations of all pollutants within the given combination (comparing concentrations at the 75^th^ percentile with those at the 25^th^ percentile). To evaluate model misspecification (including unmeasured and residual confounding) for the multipollutant joint effects models that contained interaction terms, we estimated joint associations for the combination pollutants 1 day after the ED visit admit date (while retaining the lag 0–2 pollutant concentrations as predictors) [23].

In sensitivity analyses, to test the impact of errors in air pollutant concentration estimates, we re-fit the single pollutant models after using the PM_2.5_/PM_10_ ratio to filter improbable PM_10_ concentration estimates that arise due to the relative sparseness of the PM_10_ monitoring network. Specifically, we dropped PM_10_ concentrations when the PM_2.5_/PM_10_ ratio was >1.2 or < 0.1, leading to 0.6% missing data.

## Results

Analyses included 148,256 pediatric ED visits for asthma or wheeze, 90,063 ED visits for pneumonia, 84,597 ED visits for bronchitis, 422,268 ED visits for otitis media, and 744,942 ED visits for URI in Georgia from 2002 to 2008. Descriptive statistics of the 3-day moving average pollutant concentrations and meteorological parameters for the 742 ZIP code areas in Georgia are presented in Table 1. Spearman correlation coefficients between the 3-day moving average pollutant concentrations at the ZIP code level are presented in Table 2. Air pollutants from the same sources or having similar atmospheric processes were correlated. For example, Spearman correlation coefficients between the traffic pollutants (CO, NO_2_, EC, OC) ranged between 0.32 and 0.87, and we observed moderate correlations between secondary pollutant concentrations, e.g., O_3_ and SO_4_^2−^ (*r* = 0.61) and O_3_ and NH_4_^+^ (*r* = 0.53).

**Table 1:** Three-day moving average^a^ ambient air pollutant concentrations, temperature, and humidity

Pollutant	Mean (SD)	Range	IQR^b^	25^th^ percentiles	75^th^ percentiles
1-hr max CO (ppm)	0.36 (0.26)	0.06–4.47	0.22	0.20	0.42
1-hr max NO_2_ (ppb)	9.22 (9.62)	0.07–73.88	9.33	2.70	12.03
8-hr max O_3_ (ppb)	42.1 (12.6)	5.4–106.1	18.5	32.4	50.9
1-hr max SO_2_ (ppb)	6.12 (5.18)	−0.06–112.80	5.60	2.52	8.12
24-hr avg. PM_10_ (μg/m^3^)	22.5 (8.9)	5.5–198.1	11.5	16.0	27.6
24-hr avg. PM_2.5_ (μg/m^3^)	13.2 (5.7)	2.4–86.4	6.9	9.2	16.1
24-hr avg. EC (μg/m^3^)	0.66 (0.46)	0.03–7.79	0.48	0.35	0.83
24-hr avg. OC (μg/m^3^)	2.52 (1.25)	0.26–39.87	1.45	1.65	3.09
24-hr avg. NH_4_^+^ (μg/m^3^)	1.17 (0.64)	0.10–5.84	0.73	0.72	1.45
24-hr avg. NO_3_^–^(μg/m^3^)	0.51 (0.45)	0.02–5.70	0.51	0.19	0.70
24-hr avg. SO_4_^2−^ (μg/m^3^)	3.95 (2.30)	0.46–22.55	2.72	2.28	5.00
Relative humidity (%)	60.2 (10.7)	21.4–93.2	14.8	52.8	67.6
Temperature (°C)	294.6 (8.2)	266.4–313.3	13.6	288.1	301.7

^a^The 3-day moving average was calculated for 742 ZIP code areas in Georgia during 2002–2008 (n = 1897294)

^b^IQR was calculated as the difference between the 25^th^ and 75^th^ percentile of the 3-day moving average

**Table 2:** Spearman correlation coefficients for 3-day moving average ambient air pollutant concentrations in Georgia, 2002- 2008^a^

	CO	NO_2_	O_3_	SO_2_	PM_10_	PM_2.5_	EC	OC	NH_4_^+^	NO_3_^−^
CO	1									
NO_2_	0.87	1								
O_3_	−0.15	−0.12	1							
SO_2_	0.56	0.59	−0.03	1						
PM_10_	0.05	0.03	0.68	0.03	1					
PM_2.5_	0.26	0.22	0.61	0.21	0.88	1				
EC	0.81	0.76	0.01	0.53	0.31	0.45	1			
OC	0.45	0.32	0.35	0.32	0.62	0.69	0.71	1		
NH_4_^+^	0.17	0.17	0.53	0.16	0.74	0.87	0.28	0.40	1	
NO_3_^−^	0.42	0.36	−0.39	0.45	−0.21	−0.03	0.39	0.27	−0.05	1
SO_4_^2−^	0.05	0.05	0.61	0.06	0.77	0.85	0.15	0.33	0.93	−0.28

^a^The 3-day moving average ambient air pollutant concentrations were calculated for 742 ZIP code areas in Georgia during 2002–2008 (n = 1897294)

Associations between 3-day moving average air pollutant concentrations and the five health outcomes from single pollutant models are shown in Table 3. Estimated ORs were above the null for nearly all of the single-pollutant models, though some were not statistically significant. However, given the strong correlation of certain pollutants with one another, it is likely that several of these associations are confounded by correlated pollutants.

**Table 3:** Adjusted odds ratios and 95% confidence intervals from single-pollutant models^a^

	Asthma or Wheeze		Pneumonia		Bronchitis		Otitis Media		URI	
Pollutant	OR	95% CI	OR	95% CI	OR	95% CI	OR	95% CI	OR	95% CI
CO	1.008	(1.002, 1.015)	1.015	(1.005, 1.025)	1.037	(1.024, 1.052)	1.012	(1.008, 1.017)	1.016	(1.012, 1.019)
NO_2_	1.006	(0.995, 1.018)	1.006	(0.991, 1.021)	1.024	(1.005, 1.044)	1.016	(1.009, 1.023)	1.023	(1.017, 1.028)
O_3_	1.025	(1.007, 1.042)	1.040	(1.015, 1.064)	1.027	(1.001, 1.055)	1.021	(1.010, 1.032)	1.036	(1.028, 1.044)
SO_2_	1.008	(1.000, 1.015)	1.006	(0.996, 1.016)	1.001	(0.989, 1.014)	1.003	(0.998, 1.008)	1.005	(1.001, 1.009)
PM_10_	1.037	(1.025, 1.050)	1.025	(1.009, 1.041)	1.037	(1.020, 1.054)	1.011	(1.004, 1.019)	1.030	(1.025, 1.036)
PM_2.5_	1.031	(1.021, 1.041)	1.021	(1.008, 1.035)	1.032	(1.018, 1.047)	1.011	(1.005, 1.017)	1.025	1.021, 1.030
EC	1.014	(1.007, 1.022)	1.016	(1.005, 1.027)	1.042	(1.028, 1.056)	1.012	(1.007, 1.017)	1.024	(1.021, 1.028)
OC	1.017	(1.008, 1.026)	1.018	(1.007, 1.029)	1.028	(1.016, 1.040)	1.012	(1.007, 1.017)	1.022	(1.018, 1.026)
NH_4_^+^	1.019	(1.010, 1.027)	1.013	(1.001, 1.026)	1.017	(1.003, 1.031)	1.006	(1.000, 1.011)	1.016	(1.012, 1.020)
NO_3_^−^	1.017	(1.006, 1.029)	1.008	(0.995, 1.021)	1.027	(1.012, 1.041)	0.996	(0.990, 1.003)	1.012	(1.007, 1.017)
SO_4_^2−^	1.022	(1.012, 1.032)	1.021	(1.006, 1.036)	1.014	(0.998, 1.030)	1.010	(1.004, 1.017)	1.018	(1.013, 1.023)

^a^ ORs were for interquartile range increases in 3-day moving average ambient air pollutant concentrations (units present in Table 1) and emergency department visits among children, Georgia, 2002–2008

**Fig. 1:**
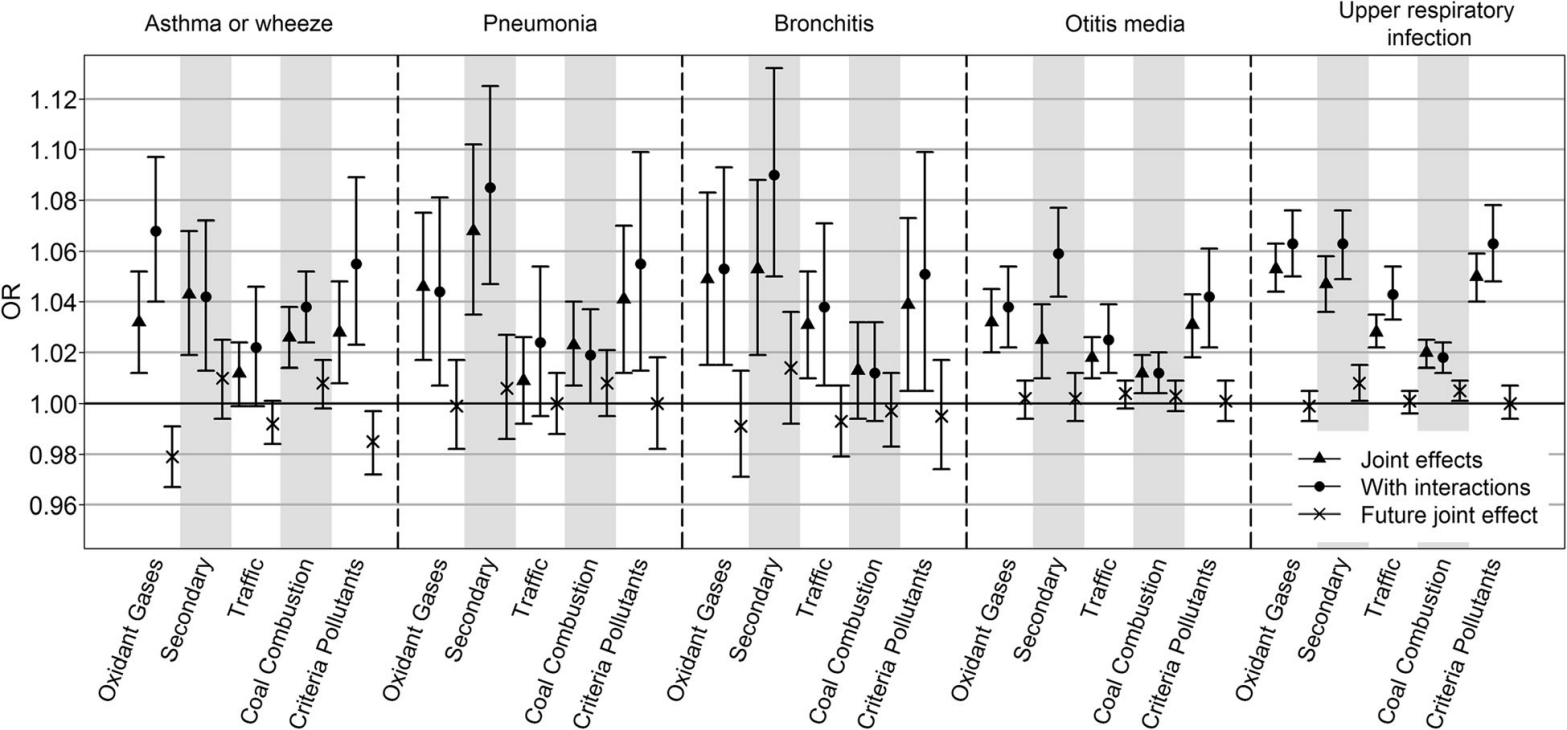
Joint effects of pollutant combinations estimated from multipollutant models. OR estimates comparing air pollutant concentrations at the 75^th^ percentile with the 25^th^ percentile for each air pollutant combination from multipollutant models without interaction (*triangle*), each air pollutant combination from multipollutant models with interactions (*circle*), and from multipollutant models (no interaction model) including future (tomorrow’s) air pollutant combinations when controlling the 3-day moving average (lag 0-1-2) of current air pollution (*cross*) were shown

The joint effect estimates from multipollutant models with and without interactions are shown in Fig. 1 (point estimates and 95% confidence intervals are provided in Additional file 1). Broadly, including first-order interactions between the pollutants tended to improve model fit, with more than half of the p-values for the likelihood ratio test for the interaction terms less than 0.05 (Additional file 1); adding interactions often increased the joint effect estimates (Fig. 1). For example, the OR estimate for the association between “oxidant gases” and asthma or wheeze and the OR estimate for the association between “secondary pollutants” and otitis media more than doubled after adding first order interactions in the models. Other OR estimates, conversely, changed little when interactions were added to the multipollutant models. In most cases the joint effects (even with interactions) were smaller than the exponentiated sum of the regression coefficients from the single pollutant models (Additional file 2).

The “secondary pollutants” had the highest magnitude OR estimates with pediatric ED visits for all health outcomes, except for asthma or wheeze, for which the OR estimate of the highest magnitude was for “oxidant gases.” The highest magnitude OR for “traffic pollutants” and “criteria pollutants” was with ED visits for URI, whereas the highest magnitude OR for “coal combustion pollutants” was with ED visits for asthma or wheeze. With few exceptions, associations of future pollutant concentrations with current ED visits were close to the null (Fig. 1) and therefore did not indicate residual confounding or gross model misspecification. Exceptions were the negative joint effects of future oxidant pollutants and future criteria pollutants for asthma or wheeze, and the positive joint effects of future secondary pollutants and future coal combustion pollutants for URI.

In sensitivity analyses, compared with OR estimates from single pollutant models using all the air pollutant concentration estimates, OR estimates from models after filtering extreme PM_10_ concentrations based on the PM_2.5_/PM_10_ ratio remained roughly the same. Estimated ORs were above the null for asthma or wheeze (OR: 1.038, 95% CI (1.026, 1.050)), pneumonia (OR: 1.023, 95% CI (1.007, 1.039)), bronchitis (OR: 1.033, 95% CI (1.016, 1.050)), otitis media (OR: 1.010, 95% CI (1.003, 1.018)), and URI (OR: 1.030, 95% CI (1.024, 1.035)).

## Discussion

In this study, we analyzed pediatric ED visits from 2002 to 2008 in relation to multiple ambient air pollutants throughout the state of Georgia. By using ambient air pollutant concentration data from CMAQ model simulations that were fused with ground measurements, the data gap of ground air pollution measurements was filled, and this allowed us to extend our study region to cover all of Georgia and analyze a large number of ED visits to support multipollutant epidemiological models. The estimated joint effects from multipollutant models indicated that the oxidant gases (O_3_, NO_2_, and SO_2_), the secondary pollutants (O_3_ and PM_2.5_ components SO_4_^2−^, NO_3_^−^, NH_4_^+^), and the criteria pollutants (O_3_, CO, NO_2_, SO_2_, and PM_2.5_) were significantly associated with increased risk for all five health outcomes. The traffic pollutants (CO, NO_2_, EC, and OC) and the coal combustion pollutants (SO_2_ and SO_4_^2−^) showed weak to moderate adverse health associations.

Comparisons between point estimates from single pollutant models and multipollutant models suggested that the point estimates from single pollutant models were affected by correlated air pollutants and may be biased due to confounding by co-pollutants. OR estimates of oxidant gases and health outcomes changed slightly between single pollutant models and multipollutant models (Additional file 2), possibly due to the relatively weak correlation among these pollutants (O_3_, NO_2_, and SO_2_). On the contrary, OR estimates of traffic pollutants (CO, NO_2_, EC, and OC) that are strongly correlated changed obviously between single pollutant models and multipollutant models (Additional file 2), indicating confounding. We note that the change in OR estimates when adding more pollutants in the models may also result from the differential measurement errors across pollutants. Thus, direct comparisons of effect estimates of specific pollutants from the multipollutant model and the single pollutant model may be misleading. Previous studies reported that in multipollutant models, the exposure-outcome relationships became weaker relative to those in single pollutant models [14, 24, 25]. In a study of air pollution and hospital admissions for respiratory diseases in Italy, Fusco et al. [24] found that the health associations of NO_2_ and O_3_ with respiratory conditions, acute respiratory infections, and asthma in children in multipollutant models were decreased relative to those in single pollutant models. Jalaludin et al. [25] reported that the health effects estimated for individual air pollutants decreased in two-pollutant models in their study of air pollution and pediatric ED visits for asthma in Australia. Consistent with these previous studies, we found that the exponentiated sum of regression coefficients from single pollutant models was larger than the corresponding joint effects from multipollutant models (Additional file 2) for most pollutant combinations and outcomes.

We observed increases in several estimated joint effects after adding first order multiplicative interactions between air pollutants, perhaps suggesting complex mechanisms of air pollution mixture exposure associated health responses. Synergism between pollutants has been reported by both laboratory studies as well as epidemiological studies [26]. A limitation of adding pollutant cross-product terms to multipollutant models is there is often limited statistical power to detect interaction effects. However, the 7-year fusion pollutant concentration estimates for the entire state of Georgia enabled us to analyze a large number of health events and assess multipollutant models with interactions. Some previous studies have also reported evidence for nonlinearities in air pollution health effects. Parametric nonlinear models [14] as well as nonparametric nonlinear models, e.g., generalized additive models [27], have been used to assess health effects of air pollution. We did not investigate non-linearity of dose-response in our study.

Several previous studies have associated health effects with air pollutant sources and properties. For example, previous studies reported that PM_2.5_ from distinct sources was associated with differential risk in daily mortality [28] and ED visits for cardiovascular and respiratory disease [29, 30]. In our study, we grouped air pollutants, including various PM_2.5_ components, in combinations based on their atmospheric processes and sources. We noticed considerable heterogeneity in joint effect estimates for each health outcome across pollutant mixtures: the secondary pollutants showed the strongest associations with all five health outcomes, while the coal combustion pollutants and the traffic pollutants showed weaker health associations. The relatively weak health effect estimates of the coal combustion pollutants may be related to the considerable measurement error in SO_2_ concentration estimates in our study region.

Otitis media has been observed to be associated with air pollution exposures in previous studies. One study reported that long-term exposure of PM_2.5_, EC, and NO_2_ was associated with increased incidence of otitis media [31]. Another reported that in Canada, increases in daily CO, O_3_, and NO_2_ concentrations were associated with elevated ED visits for otitis media year-round, and increases in daily PM_10_ concentrations were associated with elevated ED visits for otitis media in warm months [32]. No significant associations between otitis media ED visits and SO_2_ or PM_2.5_ were reported in that study, with OR estimates close to null [32]. In our study, we observed positive associations with CO, NO_2_, O_3_, PM_2.5_ and EC for otitis media in single pollutant models, whereas SO_2_ showed a very weak association (OR: 1.003, 95% CI (0.998, 1.008)). We also observed associations between all multipollutant combinations and ED visits for otitis media, with the secondary pollutants showing the strongest association (OR:1.059, 95% CI (1.042, 1.077)) and the coal combustion pollutants showing the weakest association (OR: 1.012, 95% CI (1.004, 1.020)).

The large number of ED visits analyzed in our study made it possible to precisely estimate joint health effects from multipollutant models. However, the fusion pollutant concentration estimates have prediction errors that varied across pollutants in space and time and these errors were not propagated into the epidemiologic models. According to the Friberg et al. evaluation analysis, during the study period (2002–2008), 88% of the variance in ozone concentrations was captured by the fused data, but only 14% of the variance in SO_2_ concentrations was captured [17]. This variability in prediction error is due to differences in pollutant sources and atmospheric processes. For example, the ability of the CMAQ model to simulate spatiotemporal variance of SO_2_ and PM_10_ is limited due to impact of SO_2_ plumes and biogenic PM_10_ sources [17]. In addition, different air pollutants are measured at different locations and with variable frequency, e.g., total PM_2.5_ concentrations were measured at more stations than were PM_2.5_ components, and ozone is measured mostly in summer. Most of the monitors for the PM_2.5_ components operate only once every 3 days. Some rural areas, like southern Georgia, are not covered by the ground-based air monitoring network. Thus, the ground measurements available to calibrate CMAQ simulations varied across pollutants and regions. The differential measurement errors across pollutants may contribute to variability in health effect estimates when multiple pollutants are included in the same model. These pollutant measurement error issues may also be responsible for the low health effect estimates obtained for SO_2_ and coal combustion pollutants.

One limitation of our study is misclassification of disease. We used primary ICD-9 codes to classify ED visits; however, diagnostic and coding practices can differ across hospitals, and misclassification of pediatric conditions is likely [33]. Nonetheless, disease-specific seasonal patterns were observed in the data, with ED visits for URI peaking in winter and ED visits for asthma peaking in September/October, which is consistent with previous literature [34]. Another issue is misclassification of exposure related to the use of ZIP codes. The slight modifications of ZIP codes over time, and the potential for inconsistencies between the home address ZIP code and where the child was actually residing may lead to misclassification of exposure; however, changes in ZIP code often happen locally (e.g., one ZIP code is split into two), and children’s activities will often be close to their home. Thus, for secondary pollutants that are distributed smoothly across space, the estimated health associations are unlikely to be significantly affected by this source of exposure measurement error; however, for primary pollutants that are spatially more heterogeneous, the spatial variability of exposure may be an important source of exposure measurement error, especially when local point emission sources exist [35, 36].

## Conclusions

In this study we used air pollutant concentration data from CMAQ simulations that were fused with ground measurements to model acute joint health effects of air pollutant combinations in a study of pediatric ED visits throughout Georgia. Our results suggest that air pollutant mixtures are associated with elevated risk of pediatric ED visits for asthma or wheeze, bronchitis, pneumonia, otitis media and URI. The joint effects from multipollutant models, even with interactions, were mostly smaller than the exponentiated sum of the regression coefficients from the single pollutant models, reflecting positive confounding in the single pollutant models; thus, estimating multipollutant joint effects by summing single pollutant health associations may lead to positive bias. Joint effects estimated from models that included first-order multiplicative interactions between air pollutants were frequently elevated, perhaps suggesting synergism between pollutants. Our analysis demonstrates the value of employing model-estimated air pollution estimates in epidemiological analyses and supports a multiple pollutant approach for health effects assessment.

## Additional files

Additional file 1: Joint effects for interquartile range increases in 3-day moving average of multiple ambient air pollutant concentrations from multipollutant models without and with first order interactions. (PDF 215 kb)
Additional file 2: Single pollutant effects for interquartile range increases in 3-day moving average from single pollutant models and from multipollutant models controlling for other pollutants without interaction. (DOCX 24 kb)

## Abbreviations

CMAQ: Community multi-scale air quality
EC: Elemental carbon
ED: Emergency department
ICD-9: International classification of diseases, 9th revision
IQR: Interquartile range
OC: Organic carbon
OR: Odds ratios
PM_10_: Particulate matter with an aerodynamic diameter of 10 μm or less
PM_2.5_: Particulate matter with an aerodynamic diameter of 2.5 μm or less
SD: Standard deviation
URI: Upper respiratory tract infection
