# Supplementary material for: Pediatric emergency department visits and ambient Air pollution in the U.S. State of Georgia: a case-crossover study
Source: Environ Health. 2016 Nov 25;15:115. doi: 10.1186/s12940-016-0196-y (PMC5124302; doi:10.1186/s12940-016-0196-y)
Supplement: Additional file 1: — Joint effects for interquartile range increases in 3-day moving average of multiple ambient air pollutant concentrations from multipollutant models without and with first order interactions. (PDF 215 kb) [file 12940_2016_196_MOESM1_ESM.pdf]

Additional file 1. Joint Effects for Interquartile Range Increases in 3-Day Moving Average of Multiple Ambient Air Pollutant Concentrations from Multipollutant Models without and with First Order Interactions.

Pollutant Combinations	Asthma or Wheeze		Pneumonia		Bronchitis		Otitis Media		URI	
	OR	95% CI	OR	95% CI	OR	95% CI	OR	95% CI	OR	95% CI
**Oxidant Gases (O_3, NO_2, and SO_2)**										
Without Interactions	1.032	(1.012, 1.052)	1.046	(1.017, 1.075)	1.049	(1.015, 1.083)	1.032	(1.020, 1.045)	1.053	(1.044, 1.063)
With Interactions	1.068[a]	(1.040, 1.097)	1.044	(1.007, 1.081)	1.053	(1.015, 1.093)	1.038[a]	(1.022, 1.054)	1.063[a]	(1.050, 1.076)
**Secondary Pollutants (O_3, SO_4^{2-}, NO_3^-, and NH_4^+)**										
Without Interactions	1.043	(1.019, 1.068)	1.068	(1.035, 1.102)	1.053	(1.019, 1.088)	1.025	(1.010, 1.039)	1.047	(1.036, 1.058)
With Interactions	1.042[a]	(1.013, 1.072)	1.085[a]	(1.047, 1.125)	1.090[a]	(1.050, 1.132)	1.059[a]	(1.042, 1.077)	1.063[a]	(1.049, 1.076)
**Traffic Pollutants (CO, NO_2, EC, and OC)**										
Without Interactions	1.012	(0.999, 1.024)	1.009	(0.992, 1.026)	1.031	(1.010, 1.052)	1.018	(1.010, 1.026)	1.028	(1.022, 1.035)
With Interactions	1.022[a]	(0.999, 1.046)	1.024	(0.995, 1.054)	1.038	(1.007, 1.071)	1.025[a]	(1.012, 1.039)	1.043[a]	(1.033, 1.054)
**Coal Combustion Pollutants (SO_2 and SO_4^{2-})**										
Without Interactions	1.026	(1.014, 1.038)	1.023	(1.007, 1.040)	1.013	(0.994, 1.032)	1.012	(1.004, 1.019)	1.020	(1.014, 1.025)
With Interactions	1.038[a]	(1.024, 1.052)	1.019	(1.000, 1.037)	1.012	(0.993, 1.032)	1.012	(1.004, 1.020)	1.018	(1.012, 1.024)
**Criteria Pollutants (O_3, CO, NO_2, SO_2, and $PM_{2.5}$)**										
Without Interactions	1.028	(1.008, 1.048)	1.041	(1.012, 1.070)	1.039	(1.005, 1.073)	1.031	(1.018, 1.043)	1.050	(1.040, 1.059)
With Interactions	1.055[a]	(1.023, 1.089)	1.055	(1.013, 1.099)	1.051[a]	(1.005, 1.099)	1.042[a]	(1.022, 1.061)	1.063[a]	(1.048, 1.078)

[a] p-value of likelihood ratio test less than 0.05. The p-value was calculated from likelihood statistics based on chi-square distribution and the likelihood statistics were the change of -2log likelihood between models with and without interaction terms.
