# Supplementary material for: Pediatric emergency department visits and ambient Air pollution in the U.S. State of Georgia: a case-crossover study
Source: Environ Health. 2016 Nov 25;15:115. doi: 10.1186/s12940-016-0196-y (PMC5124302; doi:10.1186/s12940-016-0196-y)
Supplement: Additional file 2: — Single pollutant effects for interquartile range increases in 3-day moving average from single pollutant models and from multipollutant models controlling for other pollutants without interaction. (DOCX 24 kb) [file 12940_2016_196_MOESM2_ESM.docx]

Additional file 2. Single Pollutant Effects for Interquartile Range Increases in 3-Day Moving Average from Single Pollutant Models and from Multipollutant Models Controlling for Other Pollutants Without Interaction.

| Pollutant | Asthma or Wheeze | | Pneumonia | | Bronchitis | | Otitis Media | | URI | |
| --- | --- | --- | --- | --- | --- | --- | --- | --- | --- | --- |
|  | OR | 95% CI | OR | 95% CI | OR | 95% CI | OR | 95% CI | OR | 95% CI |
| **Oxidant Gases (O_3_, NO_2_, and SO_2_)** | | | | | | | | | | |
| O_3_ ^a^ | 1.025 | (1.007, 1.042) | 1.040 | (1.015, 1.064) | 1.027 | (1.001, 1.055) | 1.021 | (1.010, 1.032) | 1.036 | (1.028, 1.044) |
| O_3_ ^b^ | 1.024 | (1.006, 1.042) | 1.039 | (1.015, 1.064) | 1.027 | (1.001, 1.054) | 1.018 | (1.007, 1.029) | 1.032 | (1.024, 1.041) |
| NO_2_ ^a^ | 1.006 | (0.995, 1.018) | 1.006 | (0.991, 1.021) | 1.024 | (1.005, 1.044) | 1.016 | (1.009, 1.023) | 1.023 | (1.017, 1.028) |
| NO_2_ ^b^ | 1.001 | (0.989, 1.012) | 1.002 | (0.986, 1.017) | 1.026 | (1.006, 1.047) | 1.014 | (1.006, 1.021) | 1.019 | (1.014, 1.025) |
| SO_2_ ^a^ | 1.008 | (1.000, 1.015) | 1.006 | (0.996, 1.016) | 1.001 | (0.989, 1.014) | 1.003 | (0.998, 1.008) | 1.005 | (1.001, 1.009) |
| SO_2_ ^b^ | 1.007 | (0.999, 1.015) | 1.005 | (0.994, 1.015) | 0.995 | (0.982, 1.008) | 1.000 | (0.995, 1.005) | 1.001 | (0.997, 1.005) |
| **Secondary Pollutants (O_3_, SO_4_^2-^, NO_3_^-^, and NH_4_^+^)** | | | | | | | | | | |
| O_3_ ^a^ | 1.025 | (1.007, 1.042) | 1.040 | (1.015, 1.064) | 1.027 | (1.001, 1.055) | 1.021 | (1.010, 1.032) | 1.036 | (1.028, 1.044) |
| O_3_ ^b^ | 1.014 | (0.994, 1.034) | 1.036 | (1.009, 1.063) | 1.029 | (1.000, 1.059) | 1.016 | (1.004, 1.028) | 1.031 | (1.022, 1.041) |
| SO_4_^2-^ ^a^ | 1.022 | (1.012, 1.032) | 1.021 | (1.006, 1.036) | 1.014 | (0.998, 1.030) | 1.010 | (1.004, 1.017) | 1.018 | (1.013, 1.023) |
| SO_4_^2-^ ^b^ | 1.019 | (0.982, 1.057) | 1.064 | (1.012, 1.119) | 0.989 | (0.939, 1.041) | 1.031 | (1.009, 1.054) | 0.999 | (0.983, 1.016) |
| NO_3_^-^ ^a^ | 1.017 | (1.006, 1.029) | 1.008 | (0.995, 1.021) | 1.027 | (1.012, 1.041) | 0.996 | (0.990, 1.003) | 1.012 | (1.007, 1.017) |
| NO_3_^-^ ^b^ | 1.013 | (0.999, 1.028) | 1.018 | (1.000, 1.035) | 1.025 | (1.007, 1.044) | 0.999 | (0.991, 1.007) | 1.009 | (1.003, 1.015) |
| NH_4_^+^ ^a^ | 1.019 | (1.010, 1.027) | 1.013 | (1.001, 1.026) | 1.017 | (1.003, 1.031) | 1.006 | (1.000, 1.011) | 1.016 | (1.012, 1.020) |
| NH_4_^+^ ^b^ | 0.996 | (0.964, 1.029) | 0.952 | (0.910, 0.995) | 1.009 | (0.963, 1.058) | 0.979 | (0.959, 0.998) | 1.007 | (0.992, 1.022) |
| **Traffic Pollutants (CO, NO_2_, EC, and OC)** | | | | | | | | | | |
| CO ^a^ | 1.008 | (1.002, 1.015) | 1.015 | (1.005, 1.025) | 1.037 | (1.024, 1.052) | 1.012 | (1.008, 1.017) | 1.016 | (1.012, 1.019) |
| CO ^b^ | 1.001 | (0.991, 1.012) | 1.017 | (1.002, 1.034) | 1.026 | (1.002, 1.049) | 1.009 | (1.003, 1.016) | 0.999 | (0.994, 1.005) |
| NO_2_ ^a^ | 1.006 | (0.995, 1.018) | 1.006 | (0.991, 1.021) | 1.024 | (1.005, 1.044) | 1.016 | (1.009, 1.023) | 1.023 | (1.017, 1.028) |
| NO_2_ ^b^ | 0.989 | (0.974, 1.005) | 0.979 | (0.958, 1.001) | 0.971 | (0.945, 0.998) | 1.004 | (0.994, 1.014) | 1.004 | (0.996, 1.012) |
| EC ^a^ | 1.014 | (1.007, 1.022) | 1.016 | (1.005, 1.027) | 1.042 | (1.028, 1.056) | 1.012 | (1.007, 1.017) | 1.024 | (1.021, 1.028) |
| EC ^b^ | 1.012 | (0.995, 1.028) | 1.001 | (0.978, 1.025) | 1.043 | (1.013, 1.074) | 0.996 | (0.985, 1.007) | 1.017 | (1.009, 1.026) |
| OC ^a^ | 1.017 | (1.008, 1.026) | 1.018 | (1.007, 1.029) | 1.028 | (1.016, 1.040) | 1.012 | (1.007, 1.017) | 1.022 | (1.018, 1.026) |
| OC ^b^ | 1.010 | (0.996, 1.024) | 1.012 | (0.993, 1.031) | 0.992 | (0.971, 1.012) | 1.008 | (1.000, 1.017) | 1.007 | (1.001, 1.014) |
| **Coal Combustion Pollutants (SO_2_ and SO_4_^2-^)** | | | | | | | | | | |
| SO_2_ ^a^ | 1.008 | (1.000, 1.015) | 1.006 | (0.996, 1.016) | 1.001 | (0.989, 1.014) | 1.003 | (0.998, 1.008) | 1.005 | (1.001, 1.009) |
| SO_2_ ^b^ | 1.005 | (0.998, 1.013) | 1.003 | (0.993, 1.014) | 0.999 | (0.986, 1.012) | 1.002 | (0.997, 1.006) | 1.003 | (0.999, 1.006) |
| SO_4_^2-^ ^a^ | 1.022 | (1.012, 1.032) | 1.021 | (1.006, 1.036) | 1.014 | (0.998, 1.030) | 1.010 | (1.004, 1.017) | 1.018 | (1.013, 1.023) |
| SO_4_^2-^ ^b^ | 1.021 | (1.010, 1.031) | 1.020 | (1.005, 1.035) | 1.014 | (0.998, 1.031) | 1.010 | (1.003, 1.016) | 1.017 | (1.012, 1.022) |
| **Criteria Pollutants (O_3_, CO, NO_2_, SO_2_, and PM_2.5_)** | | | | | | | | | | |
| O_3_ ^a^ | 1.025 | (1.007, 1.042) | 1.040 | (1.015, 1.064) | 1.027 | (1.001, 1.055) | 1.021 | (1.010, 1.032) | 1.036 | (1.028, 1.044) |
| O_3_ ^b^ | 1.000 | (0.981, 1.020) | 1.032 | (1.006, 1.060) | 1.007 | (0.978, 1.036) | 1.018 | (1.006, 1.030) | 1.021 | (1.012, 1.030) |
| SO_2_ ^a^ | 1.008 | (1.000, 1.015) | 1.006 | (0.996, 1.016) | 1.001 | (0.989, 1.014) | 1.003 | (0.998, 1.008) | 1.005 | (1.001, 1.009) |
| SO_2_ ^b^ | 1.005 | (0.998, 1.013) | 1.004 | (0.993, 1.014) | 0.992 | (0.979, 1.005) | 1.000 | (0.995, 1.005) | 1.000 | (0.996, 1.003) |
| NO_2_ ^a^ | 1.006 | (0.995, 1.018) | 1.006 | (0.991, 1.021) | 1.024 | (1.005, 1.044) | 1.016 | (1.009, 1.023) | 1.023 | (1.017, 1.028) |
| NO_2_ ^b^ | 0.987 | (0.972, 1.002) | 0.976 | (0.956, 0.997) | 0.982 | (0.955, 1.009) | 1.002 | (0.992, 1.012) | 1.007 | (0.999, 1.014) |
| CO ^a^ | 1.008 | (1.002, 1.015) | 1.015 | (1.005, 1.025) | 1.037 | (1.024, 1.052) | 1.012 | (1.008, 1.017) | 1.016 | (1.012, 1.019) |
| CO ^b^ | 1.003 | (0.994, 1.013) | 1.021 | (1.007, 1.035) | 1.041 | (1.020, 1.062) | 1.011 | (1.005, 1.017) | 1.008 | (1.003, 1.012) |
| PM_2.5_ ^a^ | 1.031 | (1.021, 1.041) | 1.021 | (1.008, 1.035) | 1.032 | (1.018, 1.047) | 1.011 | (1.005, 1.017) | 1.025 | (1.021, 1.030) |
| PM_2.5_ ^b^ | 1.032 | (1.020, 1.045) | 1.007 | (0.991, 1.024) | 1.018 | (1.000, 1.036) | 1.000 | (0.993, 1.007) | 1.014 | (1.008, 1.020) |

^a^ Estimated from single pollutant models

^b^ Estimated from multipollutant models
